# Higher Levels of ATGL Are Associated with Exercise-Induced Enhancement of Lipolysis in Rat Epididymal Adipocytes

**DOI:** 10.1371/journal.pone.0040876

**Published:** 2012-07-16

**Authors:** Junetsu Ogasawara, Takuya Sakurai, Takako Kizaki, Yoshinaga Ishibashi, Tetsuya Izawa, Yoshikazu Sumitani, Hitoshi Ishida, Zsolt Radak, Shukoh Haga, Hideki Ohno

**Affiliations:** 1 Department of Molecular Predictive Medicine and Sport Science, Kyorin University, School of Medicine, Tokyo, Japan; 2 Graduate School of Health and Sports Sciences, Doshisha University, Kyoto, Japan; 3 Department of Third Internal Medicine, Kyorin University, School of Medicine, Tokyo, Japan; 4 Research Institute of Sports Science, Faculty of Physical Education and Sports Science, Semmelweis University, Budapest, Hungary; 5 University of Tsukuba, Tsukuba, Ibaraki, Japan; Paris Institute of Technology for Life, France

## Abstract

**Background:**

In adipose cells, adipose triglyceride lipase (ATGL) catalyzes the first step in adipocyte triacylglyceride hydrolysis, thereby regulating both basal and hormone-stimulated lipolysis. However, little is known about the molecular mechanism(s) underlying habitual exercise-induced adaptive modulation of ATGL in white adipocytes via alteration in transcription regulator and lipolytic cofactors.

**Methodology/Principal Results:**

Male Wistar rats were randomly divided into 2 groups a sedentary control group (CG) and a habitual exercise group (EG). The EG was subjected to running on a treadmill set at 5 days per week for 9 weeks. The CG was not subjected to running on a treadmill. In the EG, levels of ATGL mRNA and protein were elevated with a significant increase in lipolysis compared with the CG, accompanied by a significant increase in associations of CGI-58 with ATGL protein. Under these conditions, an upregulation of peroxisome proliferation-activated receptorg-2 (PPARg-2) was observed. In the EG, the addition of rosiglitazone further significantly increased the levels of ATGL protein compared with the CG. However, attenuated levels of the ATGL protein in adipocytes were obtained by the addition of insulin, which is known to inhibit the expression of ATGL, in both types of groups. Actually, levels of plasma insulin were significantly reduced in the EG compared with the CG.

**Conclusions:**

These data suggest that elevated levels of ATGL are involved in the exercise-induced enhancement of lipolysis in primary adipocytes. The exact mechanism(s) underlying this phenomenon is associated, at least in part, with upregulated transcriptional activation of PPARg-2. In addition, exercise-induced lower circulation levels of insulin also correlate with habitual exercise-induced higher levels of ATGL in primary adipocytes.

## Introduction

Habitual physical exercise is beneficial to the improvement of human health by enhancing the prevention of many diseases as typified by the prevention of obesity-related metabolic syndrome. Habitual exercise enhances lipolytic responses to catecholamines in laboratory animals and humans [1]–[5], indicating that exercise is capable of stimulating lipid metabolism through modification of lipolysis-related molecules. Thus, the continuation of prolonged physical exercise plays a critical role in the positive regulation of excessive energy expense.

An increase in activities and levels of hormone-sensitive lipase (HSL) protein with translocation from the cytoplasm to the lipid droplet (LD) surface is widely accepted as the major biochemical change underlying hormone-stimulated lipolysis in adipocytes [1], [5]–[8]. However, study focusing on the white adipocytes of HSL knockout mice has revealed that no complete loss of activity in hydrolysis of triacylglycerol (TG) occurs in mice adipocytes [9], indicating other lipases are also involved in the TG degradation of adipose cells in addition to HSL. Interestingly, Zimmermann and co-workers [10] have found that adipose triglyceride lipase (ATGL) catalyzes the first step in TG hydrolysis in adipose tissue, accompanied by HSL-regulated hydrolytic degradation of TG in mammals: ATGL initially hydrolyzes TG into free fatty acid (FFA), and HSL subsequently hydrolyzes diacylglycerol substrate to produce an additional FFA and a monoacylglycerol. Indeed, a growing body of evidence has recently shown that ATGL plays a central role in the regulation of lipolysis in both basal [11] and hormone-stimulated conditions [12], [13]. However, while exercise reportedly induces upregulation of ATGL in skeletal muscle [14], and whole-body deletion of ATGL attenuates exercise performance in mice [15], at present, no study has focused on the effect that habitual exercise exerts on the molecular change of ATGL in adipocytes. Therefore, ATGL-mediated hydrolysis of TG in adipocytes is linked to lipolytic events via HSL, and the present study promotes an understanding of the habitual exercise-induced adaptive modulation of lipolytic response in adipocytes.

In the present study, we describe the manner in which exercise-induced enhancement of lipolysis is closely associated with the upregulation of ATGL following an increase in the levels and activities of PPARg-2, and also demonstrate how exercise-induced lower circulation levels of insulin regulate the levels of ATGL in primary adipocytes.

## Results

### Lipolytic Activity and the Levels of both ATGL mRNA and Protein Produced by Exercise in Primary Adipocytes

**Figure 1:**
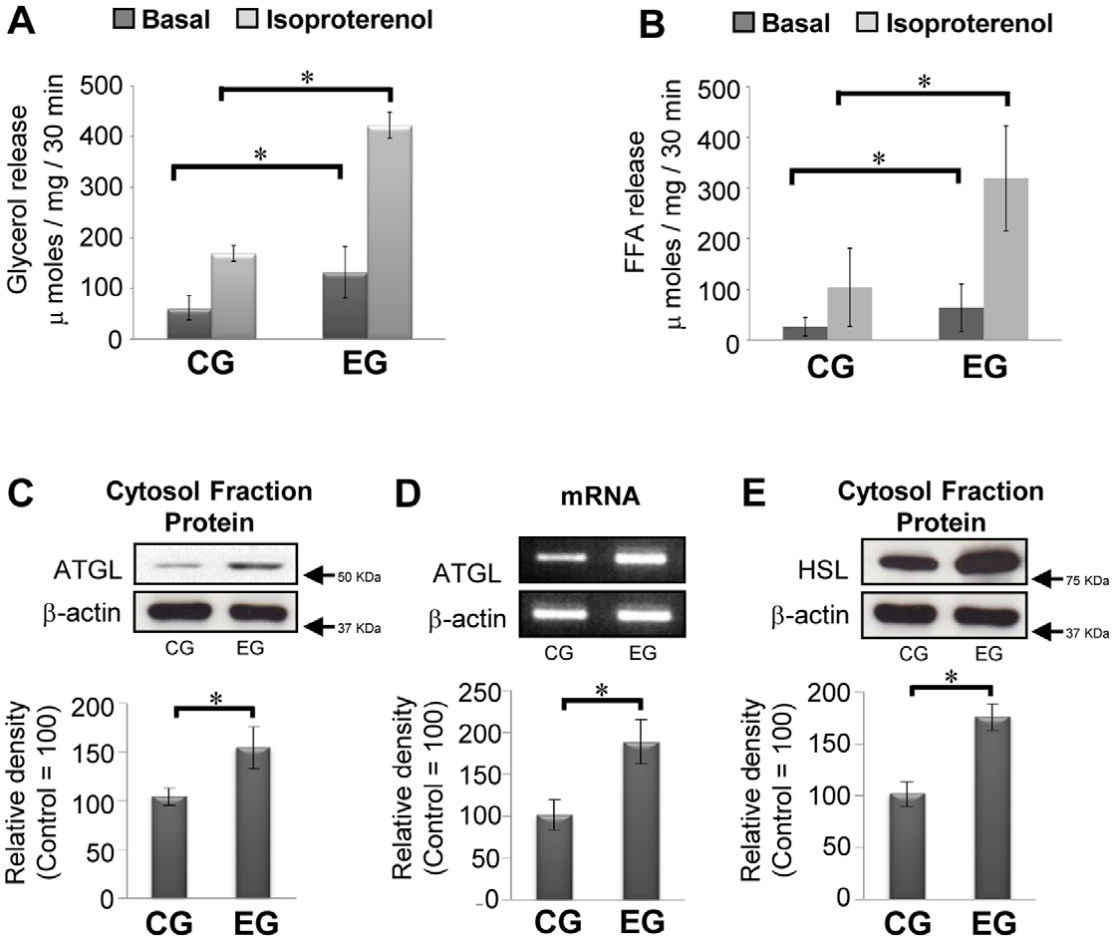
Effect of habitual exercise on lipolysis and levels of ATGL protein, mRNA and HSL protein. (A and B) Both glycerol and FFA releases, as an index of lipolysis, are shown with or without isoproterenol in adipocyte from sedentary control group (CG) and habitual exercise group (EG) rats (n = 10 for each group). (C and D) Both mRNA band and representative immunoblotting band of ATGL (upper panel) with the relative density of each band (lower panel) are shown (control  = 100, n = 10 for each group). (E) Representative immunoblotting band of unphosphorylated HSL (upper panel) with the relative density of each band (lower panel) are shown (control  = 100, n = 10 for each group). Results were representative of three independent experiments. Bars and vertical lines indicate mean ± SD. *p<0.05.

The lipolytic response in adipocytes [5] and skeletal muscles [16] was improved in the EG compared with that of the CG, and also, the overexpression of ATGL in adipocytes induced higher rates of lipolytic activity in the absence of protein kinase A (PKA)-stimulated (basal state) conditions [11]. To determine whether an exercise-induced increase in lipolysis is mediated by alterations in lipolytic activity with changes in levels of ATGL, we investigated the both glycerol and FA contents of a cell-free incubation medium and levels of ATGL. In the present study, glycerol contents were used as a principal index of lipolysis, because it has been reported that the potential for error in estimating lipolysis in adipocytes, which possess weak glycerokinase activity, by glycerol production alone are negligible [17], although the rates of FFA esterification in adipocytes are increased by hormonal stimulation, i.e., epinephrine, glucagon, adenocorticotropic hormone, and thyroid-stimulating hormone activity [18]. On the other hand, it has shown that ATGL has potential role in hydrolysis of TG to deacylglyceride (DG), which is first step of lipolysis, suggesting that alteration in levels of FFA would also be available as an index of ATGL-mediated lipolysis. As shown in Fig. 1, when compared with the CG, the both glycerol and FFA releases in the EG was significantly elevated in both basal and isoproterenol-stimulated conditions. Under these conditions, the levels of ATGL protein in EG were significantly higher than those in the CG, accompanied by elevated levels of ATGL mRNA (Fig. 1B and C). Moreover, in the EG, previously reported increases in the levels of unphosphorylated HSL protein [5] were confirmed (Fig. 1D), suggesting that adipocytes obtained in the current study acquired an adaptive character due to prolonged habitual exercise. These results indicated that upregulation of the ATGL in primary adipocytes is closely associated with habitual exercise-induced enhancement of lipolysis in primary adipocytes.

### Exercise-induced Alterations in Expression of Perilipin 1, CGI-58 and its Interaction with ATGL in Primary Adipocytes

**Figure 2:**
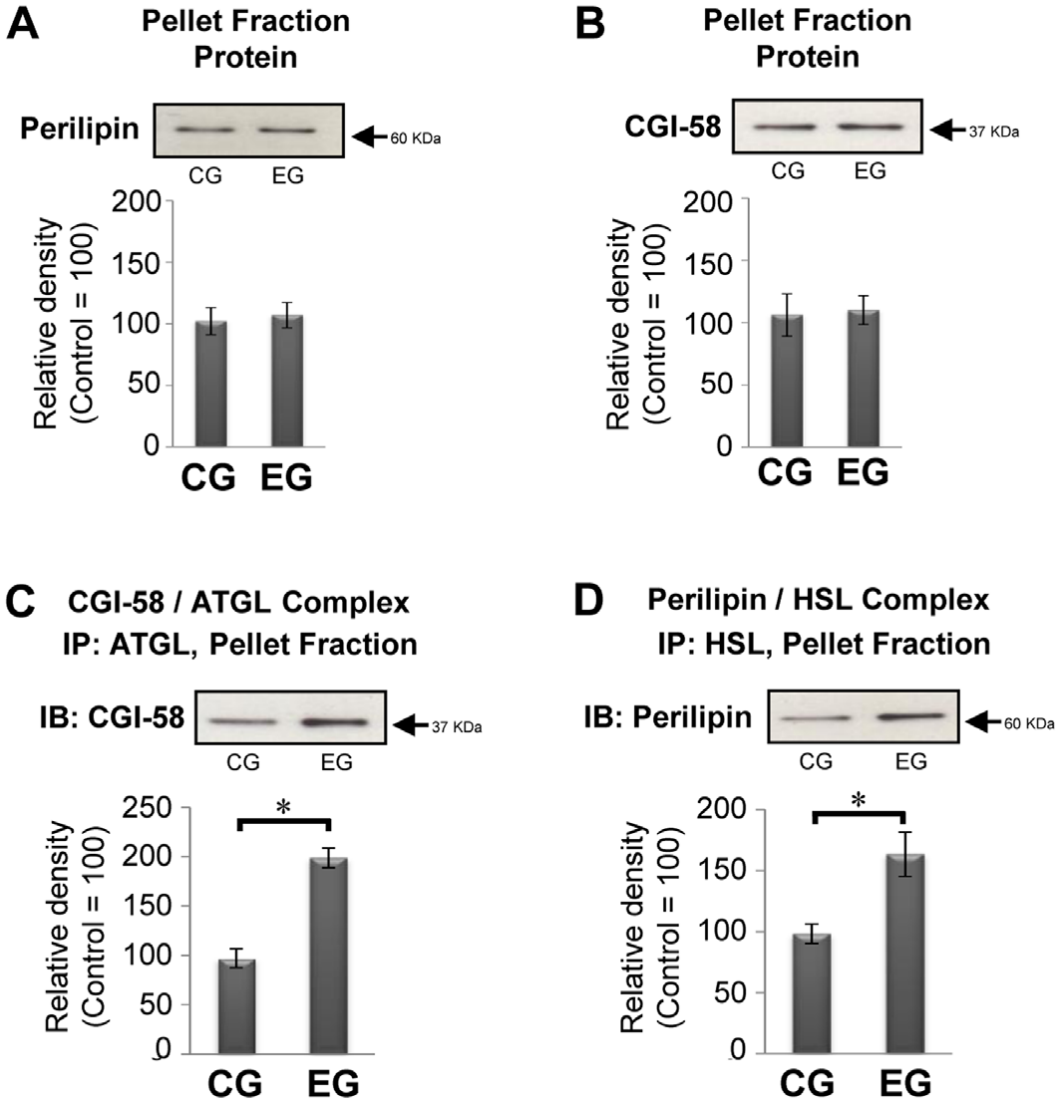
Effect of exercise on the expression of perilipin 1, CGI-58 and its interaction with ATGL and HSL in the pellet fraction. (A, B, C and D) Representative immunoblotting band (upper panel) with the relative density of each band (lower panel) are shown (control  = 100, n = 10 for each group). Bars and vertical lines indicate mean ± SD. *p<0.05. IP: immunoprecipitation; IB: immunoblotting.

There is growing evidence that both perilipin 1 and comparative gene identification-58 (CGI-58) are viewed as components of dynamic scaffold proteins that serve as a lipid droplet-associated organizing center for enzymes [19], [20]. Interestingly, the stimulated lipolysis in adipocytes releases CGI-58 from perilipin 1, in turn, CGI-58 binds to ATGL on the lipid droplet [21]. Moreover, the results obtained from our previous study demonstrated that acute exercise leads to change in associative and dissociative actions of both perilipin 1 and CGI-58 with HSL [22], suggesting that because adaptation of adipocytes in response to habitual exercise results from an integrative effect of acute exercise, habitual exercise would be capable of contributing to changes in the levels of perilipin 1 and CGI-58. As shown in Fig. 2A and B, in a pellet fraction, neither perilipin 1 nor CGI-58 levels were changed in both groups, whereas associations of CGI-58 with ATGL proteins were significantly increased in the EG (Fig. 2C). Moreover, in the EG, the interaction levels of perilipin 1 with HSL were also significantly increased (Fig. 2D), suggesting that habitual exercise alters subcellular compartmentalization of both lipases and lipolytic cofactors, thereby causing the enhancement of lipolysis. Thus, elevated levels of ATGL in the EG would be functionally associated with action of the scaffold CGI-58 protein, leading to modification of the lipid droplet surface to allow hydrolysis of TG by ATGL.

### Effect of Habitual Exercise on both Levels and Function of the PPARg-2

**Figure 3:**
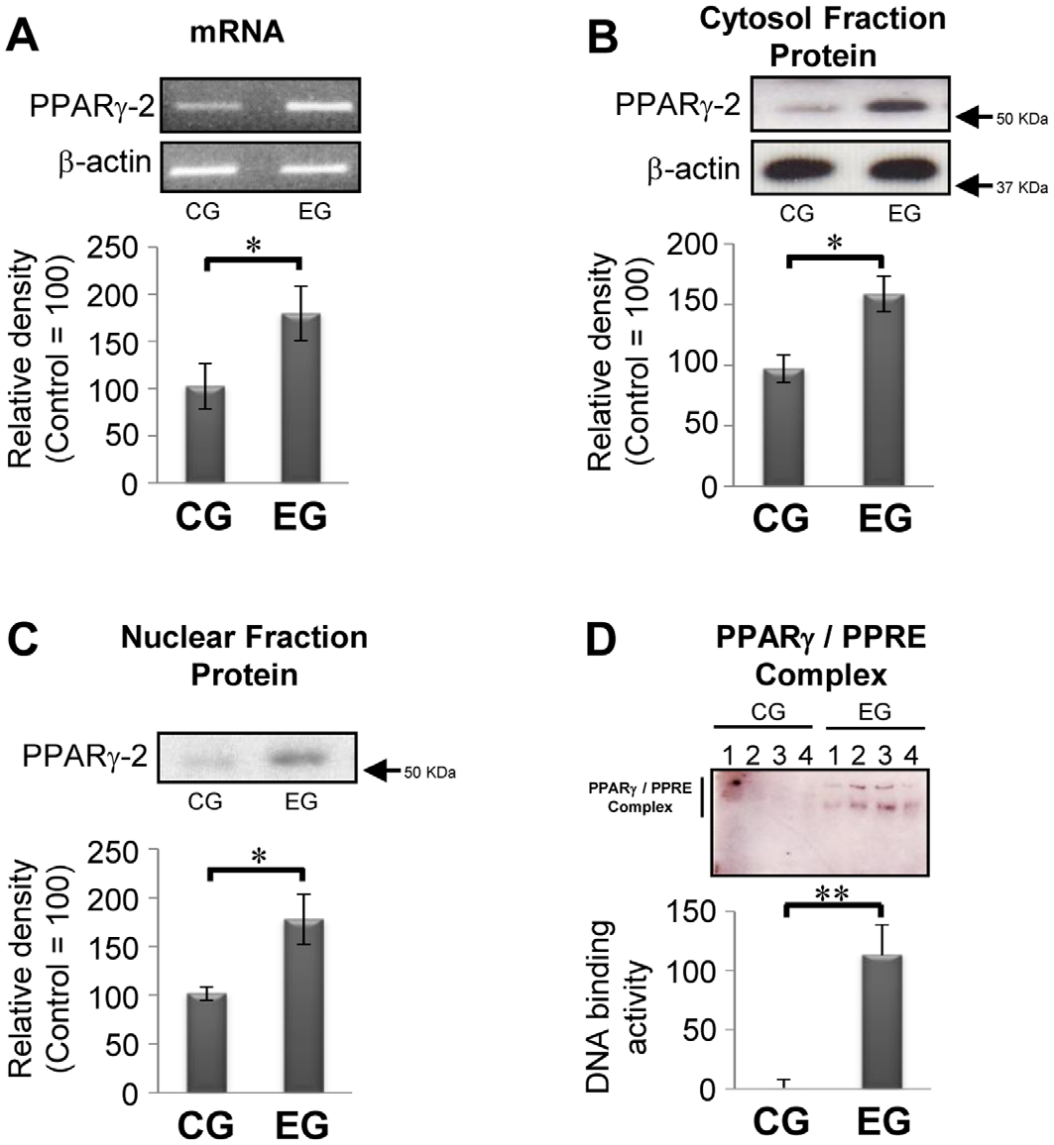
Effect of exercise on levels of PPARg -2 mRNA, protein and DNA binding activity. (A) Representative PPARg-2 mRNA and (B and C) immunoblotting data (upper panel) with the relative density of each band (lower panel) in both cytosol (B) and nuclear (C) fractions are shown (control  = 100, n = 10 for each group). (D) Activity of PPARg-2 in nuclear fraction was analyzed by EMSA (n = 4). Results were representative of three independent experiments. Bars and vertical lines indicate mean ± SD. *p<0.05 and **p<0.01. PPRE: PPAR response element.

One of the mechanisms underlying the expression of ATGL is related to stimulation by rosiglitazone, an agonist of PPARg [23], [24]. Moreover, it has been reported that levels of PPARg in adipose tissue are upregulated by habitual exercise through an increase in the activation of DNA binding [25], [26]. Hence, we next investigated the effect of habitual exercise on the expression of PPARg-2 mRNA and protein, in addition to evaluating its transcriptional activity. The levels of PPARg-2 mRNA and protein in primary adipocytes were significantly higher in the EG compared with the CG (Fig. 3A and B). Under these conditions, in a nuclear extract fraction, the level of PPARg-2 was also significantly increased in the EG compared with the CG, accompanied by elevated levels of DNA binding (Fig. 3C and D). These results suggest the possibility that exercise-induced modification of PPARg-2 is related to the upregulation of ATGL in the EG.

### Rosiglitazone Stimulates Lipolysis in Adipocytes which are Obtained by EG

**Figure 4:**
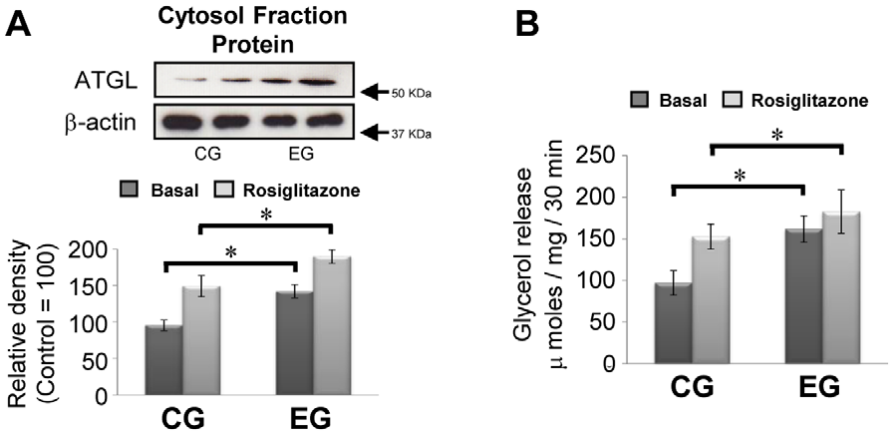
Rosiglitazone increases the levels of ATGL protein with elevated rates of the lipolysis. (A) Representative immunoblotting data (upper panel) with the relative density of each band (lower panel) are shown (control  = 100, n = 10 for each group). (B) The rate of rosiglitazone-induced lipolysis in primary adipocytes is shown (control  = 100, n = 10 for each group). Results were representative of three independent experiments. Bars and vertical lines indicate mean ± SD. *p<0.05.

Based on the results shown in Fig. 3, to confirm whether the exercise-induced enhancement of expression of ATGL was mediated through the upregulation of PPARg-2, we further investigated the effect of rosiglitazone on the levels of ATGL and lipolysis in adipocytes in both groups. As shown in Fig. 4A and B, irrespective of rosiglitazone, the results suggest that stimulation of PPARg-2 plays a role in the exercise-induced modification of ATGL expression with increasing levels of lipolysis.

### PPARg Regulates Expression of ATGL in HeLa Cells

**Figure 5:**
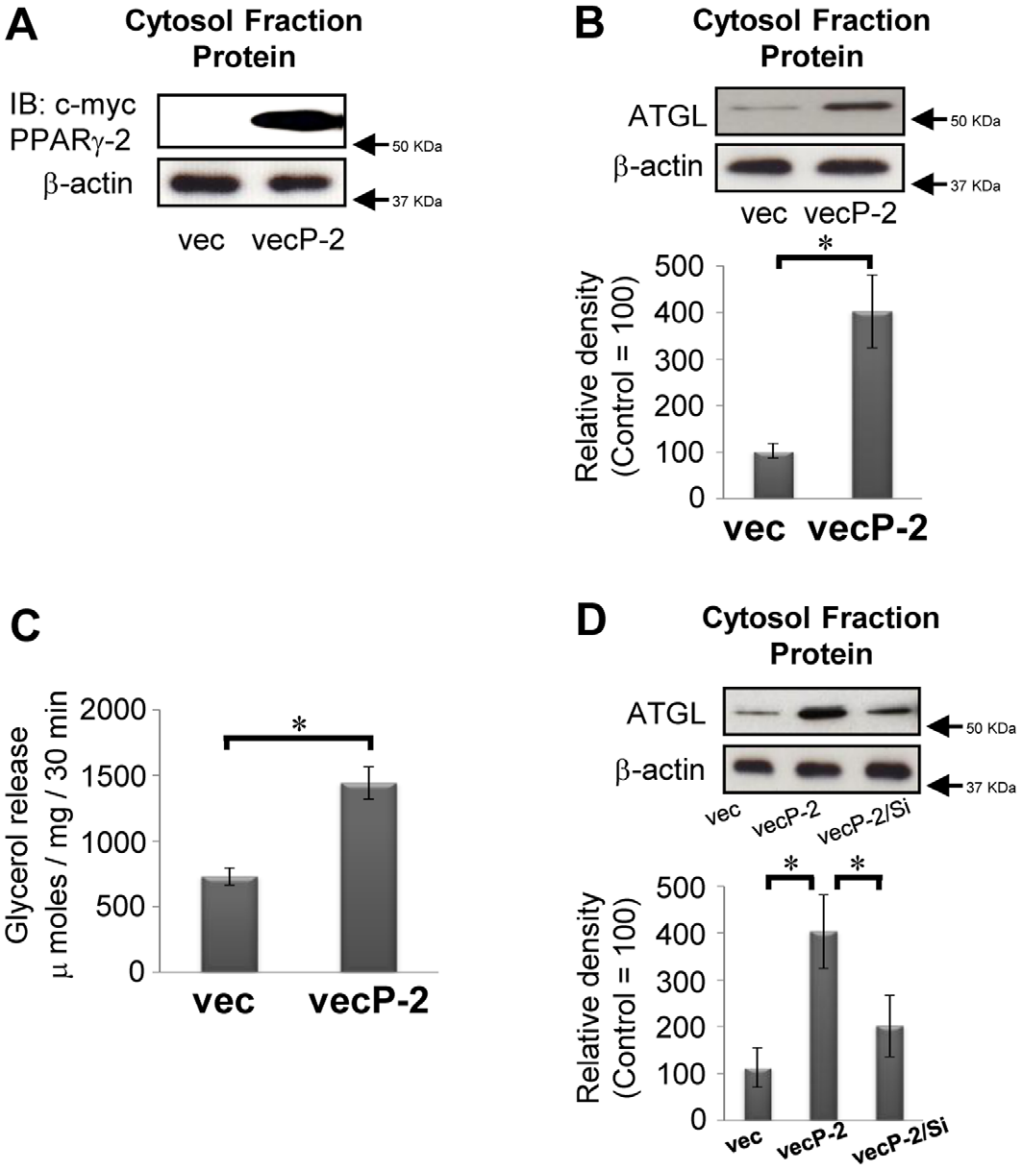
PPARg -2 translationally modifies ATGL protein in non-adipose HeLa cells. (A) Expressed PPARg-2 proteins are conformed by immunoblotting using c-myc antibody. (B and D) Representative immunoblotting data (upper panel) with the relative density of each band (lower panel) are shown (control  = 100, n = 3). (C) The rates of released glycerol into incubation medium are shown (n = 3). Results were representative of three independent experiments. Bars and vertical lines indicate mean ± SD. *p<0.05 vs. control value. vec: mock-transfected control cells; vecP-2: PPARg-2-transfected cells; vecP-2/si: co-transfected cell of both PPARg-2 vector and its siRNA. IB: immunoblotting.

It is intriguing that previous studies have shown endogenous ATGL to be localized on the external surface of lipid droplets in HeLa cells, which are a non-adipocyte cell line, and expression levels of ATGL have defined the lipid droplet size of cultured HeLa cells. Essentially, the overexpression of ATGL in HeLa cells caused a decrease in the average size of lipid droplets, whereas the knockdown of ATGL by RNA interference led to an increase in their size [27], indicating that this cell line appears to be available for models of adipose cells via transcriptional activation of PPARg. Moreover, our preliminary study showed that cell transplantation efficiency in HeLa cells was higher than that in 3T3-L1 adipocytes (data not shown). Hence, to test whether PPARg-2 has the capacity to affect the levels of ATGL, we overexpressed a c-myc-tagged version of wild-type PPARg-2 in HeLa cells. As shown in Fig. 5A, total levels of PPARg-2 protein in HeLa cells were significantly higher than in mock cells. Under these conditions, overexpression of PPARg-2 in HeLa cells caused a significant increase in levels of ATGL protein, accompanied by higher rates of glycerol release compared with transfected mock cells (Fig 5B and C). Moreover, co-transfection of both c-myc-tagged PPARg-2 and silencing of PPARg-2 demonstrated lower levels of ATGL protein compared with transfected c-myc-tagged PPARg-2 (Fig. 5D), which indicated that PPARg-2 is a transcriptional activator of ATGL, thereby enhancing turnover of triacylglycerol in ATGL-expressed cells.

### Insulin Attenuates Expression of ATGL both in vitro and in vivo

**Figure 6:**
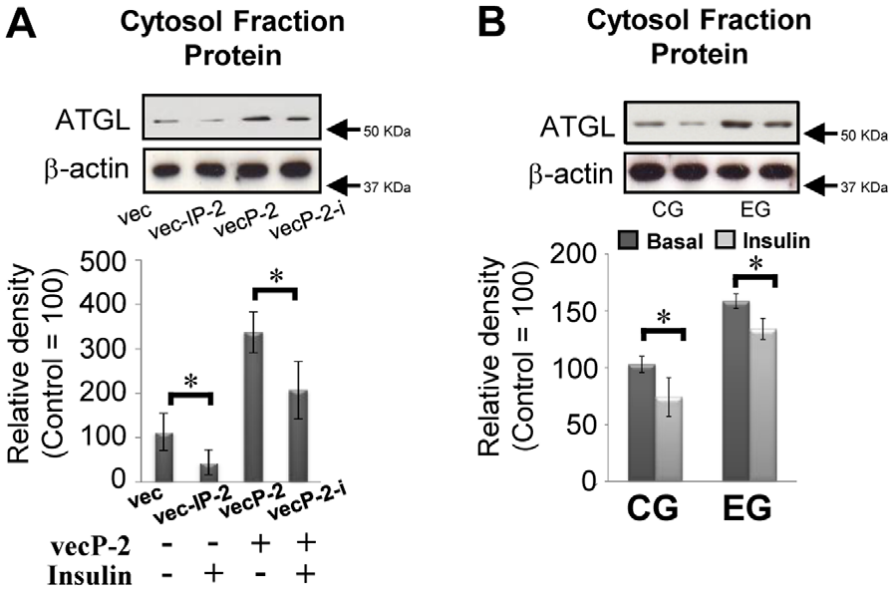
The addition of insulin attenuates the levels of ATGL protein. (A and B) Representative immunoblotting data (upper panel) with the relative density of each band (lower panel) are shown (control = 100, n = 3 and n = 10 for each group, respectively). Results were representative of three independent experiments. Bars and vertical lines indicate mean ± SD. *p<0.05. vec: mock-transfected control cells; vec-I: vec cells with insulin; vecP-2: PPARg-2-transfected cells; vecp-2-I: PPARg-2-transfected cells with insulin.

Habitual exercise is known to attenuate levels of plasma insulin in rodents [28] and humans [29]. Moreover, Kim and co-workers [30] demonstrated that insulin at 100 nM resulted in a marked decrease in ATGL transcript in 3T3-L1 adipocytes. Therefore, an exercise-induced decrease in the levels of plasma insulin might be associated with upregulation of ATGL in adipocytes. As shown in Fig. 6A, the addition of insulin definitely decreased the expression levels of ATGL in HeLa cells. Similarly, in primary adipocytes, the levels of ATGL protein were significantly reduced by the addition of insulin in both groups (Fig. 6B). Actually, levels of plasma insulin were significantly reduced in the EG compared with the CG (Table 1). Thus, low levels of insulin would also have triggered a promotion of ATGL expression in the primary adipocytes of the EG.

**Table 1:** The levels of body weight, adipose tissue weight (epididymal) and plasma insulin in both CG and EG.

	CG	EG
Body weight (g)	297.4±9.6	268.0±8.4*
A dipose tissue weight (g)	5.2±0.5	3.7±0.4*
Plasma insulin (nM)	0.53±0.04	0.34±0.01*

Values are expressed as mean ± SD.

* P<0.05 vs. CG value.

## Discussion

As we have described, habitual exercise-induced enhancement of lipolysis is closely associated with the upregulation of ATGL following an increase in the levels and DNA binding activities of PPARg-2 in primary adipocytes. Indeed, results obtained in the current study demonstrated that the addition of rosiglitazone, an agonist of PPARg-2, to primary adipocytes enhanced levels of ATGL protein with significant increase in lipolysis in both CG and EG, and that overexpression of PPARg-2 in HeLa cells led to an increase in levels of ATGL protein, whereas co-transfection of both the vector and the siRNA of PPARg-2 attenuated increased levels of ATGL. Thus, upregulation of PPARg-2 would have the capacity to modulate protein synthesis of ATGL. Moreover, in the EG, significant increases in interactions of the CGI-58 with ATGL proteins were observed compared with the CG, indicating that habitual exercise also promoted the combination of ATGL with a substrate on the lipid droplet. This concept would be supported by our results showing that the interaction of perilipin 1 with HSL was significantly increased in the EG, because CGI-58 released perilipin 1, which binds with HSL to initiate lipolysis. Finally, the addition of insulin significantly reduced the endogenous levels of ATGL protein in primary adipocytes and HeLa cells, and these phenomena were amplified in HeLa cells, which overexpressed ATGL by transfection of the PPARg-2 vector. Actually, in vivo plasma concentrations of insulin were significantly decreased in the EG compared with that in the CG. Therefore, lower circulation levels of insulin, which have an inhibitory effect on ATGL, would also be closely-involved in habitual exercise-induced augmentation of ATGL in primary adipocytes.

It is particularly notable that prolonged exercise provokes upregulation of ATGL in primary adipocytes with alteration in the localization of lipolytic cofactors, which exists (not exists) on the lipid droplets. Both CGI-58 and perilipin 1 are reportedly viewed as components of lipid droplet-associated protein [19], [20], i.e., stimulation of lipolysis in adipocytes releases perilipin 1 from CGI-58, and in turn perilipin 1 is capable of binding to activated HSL, which is accompanied by the association of CGI-58 with ATGL [21], [31]. Indeed, the results obtained from our previous study revealed that dissociation of perilipin 1 from CGI-58 and interaction of perilipin 1 with HSL are provoked during and after a single bout of exercise with enhancement of lipolysis in primary adipocytes [22], suggesting that exercise has the capacity to change the localization of the lipolytic molecules under physiological conditions. Nomura and co-workers have also demonstrated that habitual exercise enhances the levels of protein kinase A (PKA)-anchoring protein 150, which supports the binding of PKA and its substrate, thereby activating HSL in primary adipocytes [5]. Thus, alterations in the molecular behavior of the lipolytic cofactors would facilitate lipolytic responses in habitual exercise, resulting in subsequent full lipolysis. There has been no evidence that habitual exercise induces no change in the levels of perilipin 1 and CGI-58 proteins in primary adipocytes (Fig. 2A and B). Perilipin 1 null mice exhibit constitutively attenuated hormone-stimulated lipolysis [32], [33]. Therefore, the habitual exercise-induced adaptive modulation of these proteins could produce, at least in part, an increase in the binding efficiency of these proteins and lipases in primary adipocytes.

It is noteworthy that exercise-induced increases in the levels of ATGL are regulated via transcriptional activation of PPARg-2. The potential physiological effects of PPARg-2 in adipocytes are widely accepted as a master transcriptional regulator of adipocyte differentiation from pre-adipocytes to mature adipocytes [34]. Meanwhile, in mature adipocytes, little is known of another function of PPARg-2, wherein it acts as a transcription-modulating factor. Recently, several studies have demonstrated that treatment of rosiglitazone mediates induction of ATGL mRNA and protein, whereas this event is inhibited by the PPARg-specific antagonist and siRNA-mediated knockdown of PPARg-2 [30], [35]–[37]. These results indicate that PPARg-2 has the direct effect of transcriptional regulation on ATGL in mature adipose cells. Moreover, in the present study, overexpression of PPARg-2 in HeLa cells led to an upregulation in levels of ATGL, which suggests that transcriptional modification through PPARg-2 activation is a common mechanism not only in adipocytes but in ATGL-expressed cells [27]. However, it is difficult to elucidate the mechanisms underlying the exercise-induced upregulation of PPARg-2 function in primary adipocytes. It is logical to infer that increased release of fatty acids from adipocytes could be the trigger for activation of PPARg-2, because fatty acids are known to activate PPARg [38]. In fact, after a single bout of exercise, the concentration of plasma fatty acids is immediately elevated in rats [39] and humans [40]. Thus, the higher levels of plasma concentration of fatty acids, which are induced by sustained acute exercise, might play a key role in the habitual exercise-induced activation of PPARg-2 in primary adipocytes.

It is difficult to elucidate the mechanisms underlying the insulin-induced down-regulation of ATGL via transcription factors. Forkhead box protein O1 (FoxO1) reportedly transrepresses PPARg target genes via direct protein-protein interaction, and insulin induces FoxO1 phosphorylation and nuclear exportation, which prevents FoxO1-PPARg interactions and results in the rescue of the FoxO1-induced transrepression of PPARg [41]. However, in the present study, levels of plasma insulin were significantly decreased in the EG compared with the CG (Table 1). Therefore, the insulin-stimulated rescue of FoxO1-induced transrepression of PPARg might have been down-regulated in the adipocytes of the EG. Indeed, it has been shown that the insulin-induced inhibition of ATGL expression in adipocytes is caused by the direct effect of restraining the nuclear localization of FoxO1 itself rather than by the FoxO1-mediated transrepression of PPARg [42]. Consequently, lower levels of insulin in the EG could result in stored FoxO1 in the nucleus, thereby upregulating the ATGL. Thus, habitual exercise-induced upregulation of the ATGL in primary adipocytes could result from the mechanism independent of the changes in FoxO1 and FoxO1-mediated transrepression of PPARg.

ATGL-knockout mice are known to lack the ability to adjust circulating FA levels to the increased energy requirements of working muscle by a single bout of exercise [43], and twelve weeks of exercise training has significantly increased ATGL in human skeletal muscle [44]. These results suggest that ATGL activity is required for the proper energy supply of whole body metabolism during exercise. Accordingly, the results of the current study support those of previous studies: increased levels of ATGL in the EG help provide fuel for working muscles via supply of FA from adipocytes. In previous study, fasting for 72 hours has activated the expression of ATGL in human adipose tissue with insulin resistance [45]. However, a simple comparison cannot be made between fasting and exercise-induced upregulation of ATGL, although exercise fails to cause insulin resistance in adipose tissue.

In conclusion, habitual exercise exhibits the stimulatory effect of lipolysis in primary adipocytes through higher levels of ATGL. The mechanisms behind this phenomenon would involve enhanced levels and transcriptional activation of PPARg-2. Moreover, habitual exercise-induced lower levels of plasma insulin, which has been recognized in the literature, would also play a role in this mechanism.

## Materials and Methods

### Animal Care and Exercise Training Program

Five-week-old male Wistar rats (SLC, Shizuoka, Japan) were housed in groups of 2 or 3 per cage in a temperature-controlled room at 23°C with a 12∶12-h light-dark cycle. Food and water were available ad libitum. The animals were randomly divided into 2 groups: a control group (CG) (n = 10) and an exercise group (EG) (n = 10). The EG rats were subjected to running on a treadmill set at a 5-degree incline 5 days per week for 9 weeks according to a protocol previously reported [3]. The initial training intensity was 15 m/min for 20 min; thereafter, the running speed and duration were progressively elevated until, after 6 weeks, the rats ran continuously at 30 m/min for 90 min. The CG rats were not subjected to running on a treadmill. The EG rats were euthanized at 36 h after the last exercise session. The rats were anesthetized with an intraperitoneal injection of pentobarbital sodium (5 mg/100 g body weight; Abbott Labs, Abbott Park, IL). Adipose tissue was rapidly removed and isolated to adipocytes by the methods described below. The animal care committee of the Kyorin University School of Medicine approved the animal protocol.

### Preparation of Primary Adipocytes and Assay for Lipolysis

Adipocytes were isolated using a method developed by Rodbell [46]. Briefly, fat pads were minced with scissors and placed in plastic vials in buffer A (Krebs-Ringer bicarbonate solution buffered with 10 mM HEPES, pH 7.4, containing 5.5 mM glucose and 2% (w/v) fatty acid-free bovine serum albumin) with 200 nM adenosine and collagenase type1 (1 mg/ml, Worthington Biochemical, Lakewood, NJ). Collagenase digestion was performed at 37°C in a water-bath shaker. After 15 min, the contents of the vials were immediately filtered in mesh and centrifuged at 100 *g* for 1 min. The layer of floating cells was then washed 3 times with buffer A. Adipocytes were incubated in plastic vials in a total volume of 500 µl buffer A containing adenosine deaminase (0.05 mg/ml, Sigma, St. Louis, MO). After a 2 min pre-incubation, adipocytes (approximately 10^9^ cells) were incubated for 30 min again with or without 1 mM isoproterenol or 1 mM rosiglitazone [23] to investigate lipolytic responses; the cell-free incubation medium was removed and assayed for both glycerol and FFA releases as an index of lipolysis. The average number of adipocytes and glycerol contents was determined according to the method described earlier [5], [16]. The FFA content of the incubation medium was assayed by using a Free Fatty Acid Quantification Kit (BioVision Research Products, Mountain View, CA) according to the manuscript’s protocol.

### Preparation of Nuclear Extract and Electrophoretic-mobility Shift Assay (EMSA)

The nuclear extracts from primary adipocytes were prepared using a commercially available kit (Panomics, Dunbarton Circle, Fremont, CA), according to the manuscript protocol. The PPARg EMSA kit was purchased from Panomics. EMSA was performed according to the manufacturer’s protocol. Briefly, the nuclear protein and PPARg-specific probe were incubated in 10 ml of EMSA buffer at 15°C for 30 min, electrophoresed in 6% polyacrylamide gels, transferred onto nylon membranes, and UV cross-linked for 3 min. To detect the signals, membranes were incubated for 15 min with streptavidin-horseradish peroxidase conjugate in blocking reagent, and for 5 min with chemiluminescence reagent. Blots were detected using Kodak X-ray film (Kodak, Tokyo, Japan).

### Treatment of HeLa Cells

The HeLa cells were purchased from RIKEN BRC CELL BANK (RIKEN, Tsukuba, Japan). The HeLa cells were propagated in Dulbecco’s modified Eagle’s medium (DMEM) supplemented with 10% calf serum and antibiotics (50 units/ml penicillin and 50 mg/ml streptomycin). For studies of regulation of insulin (1 mM) [23], HeLa cells were incubated with both agents for 24 h, and then used for preparation of extraction of both proteins as described below.

### Preparation of Plasmid and Transfections

cDNA encoding a full open reading frame of the mouse PPARg-2 was amplified from total RNA isolated from male wistar rat epididymal adipose cells by RT (reverse transcriptase)-PCR using specific primers (5′-CGGCGCCATGGAGCTC ATGGGTGAAACTCT-3″and 5′-ATGGAGGCCCCTCGAG ATACAAGTCCTTGT-3′). The purified PCR product was directly inserted into the pCMV-myc (Clontech). pCMV-myc-PPARg-2 was created by cloning PPARg cDNA with 5′ -ECoR1 and 3′- Xho1 site down-stream and in-frame with the myc sequence in pCMV-myc. The entire PPARg-2 coding reason was included except for the stop codon. siRNA of PPARg was purchased from Santa Cruz Biotech. Inc. (Santa Cruz, CA). Plasmid and siRNA of PPARg were transfected into HeLa cells using Lipofectamine (Invitrogen) according to the manuscript’s protocol.

### Protein Extraction

Both primary adipocytes and HeLa cells were collected and washed 3 times with phosphate-buffered saline (137 mM NaCl, 8.1 mM Na_2_HPO_4_, 2.68 mM KCl, 1.47 mM KH_2_PO_4_). The cells were homogenized in ice-cold homogenization buffer (Pierce, Rockford, IL) including both protease inhibitor cocktail (Roche Diagnostics, Indianapolis, IN) and phosphatase inhibitor cocktail (Sigma, St Louis, MO), by 20 passages through a 5/8-inch, 27-gauge needle attached to a syringe maintained at 4°C. The homogenate was centrifuged at 40,000 *g* and 4°C for 30 min. The supernatant obtained was centrifuged again, and the clear sample obtained by this procedure was used as the cell extract (Cytosol fraction) for immunoblotting analysis. The resultant fat layer and pellet were resuspended and homogenized by plastic pestle in an ice-cold homogenization buffer described above with a detergent (0.2% (w/v) 3-[(3-cholamidopropyl) dimethylamino] propansulfonic acid) (Wako Osaka, Japan). The suspension was incubated on ice for 10 min and centrifuged at 40,000 *g* and 4°C for 30 min. The resultant clear supernatant below a floating lipid residue was used as a pellet fraction, which included a lipid droplet-associated protein, for immunoblotting. The samples were frozen at –80°C for later analysis.

### Immunoblotting Analysis

There was no significant difference in the protein level between cells from the different groups in each fraction (data not shown). Therefore, identical loading amounts of each sample were run on the same gel. The samples were mixed with Laemmli’s sample buffer and then placed in a heat block at 100°C for 3 min. The samples were cooled and then loaded onto a 9–12% SDS-polyacrylamide gel. After electrophoresis, the proteins were transferred onto a PVDF sequencing membrane (Millipore Corporation, Billerica, MA). The PVDF membrane was first incubated for 60 min in TBS-T (100 mM of Tris-HCl, pH 7.4, 150 mM of NaCl, and 0.1% Tween 20) containing 5% skim milk. After incubation, the PVDF membrane was incubated with a specific antibody in the TBS-T at 4°C overnight. The following antibodies were used at a 1∶1,000 dilution: PPARg, CGI-58/abhd5, perilipin A/perilipin 1, b-actin (Abcam, Cambridge, UK), HSL, ATGL (Cell Signaling Technology, Inc., Danvers, MA), c-myc (Clontech, Mountain View, CA). After washing, the membranes were incubated for 60 min with anti-rabbit and anti-goat immunoglobulin G (1∶2,000 dilution)-conjugated horseradish peroxidase antibody (Dakocytomation, Glostrup, Denmark). The membranes were washed, and the immunoreactive bands were detected using the ECL system (GE Healthcare, Buckinghamshire, UK) by Kodak X-ray film (Kodak, Tokyo, Japan).

### Immunoprecipitation

The pellet fractions lysate, a total of 150 mg of protein, were incubated with rabbit normal IgG (Santa Cruz Biotech. Inc., Santa Cruz, CA) and proteinA/G agarose beads (Santa Cruz Biotech. Inc., Santa Cruz, CA) at 4°C for 30 min. After centrifugation at 2500 *g* and 4°C for 5 min, the supernatant were collected and incubated with primary antibody to HSL or ATGL (0.5 mg, respectively) and protein A/G agarose beads at 4°C for overnight. After centrifugation at 2500 *g* and 4°C for 5 min, beads were collected and washed 4 times in ice-cold PBS. Pelleted beads were mixed with 20 ml of Laemmli’s sample buffer and boiled at 100°C for 5 min. After centrifugation at 2500 *g* and 4°C for 5 min, a total of 15 ml of carefully aspirated supernatant were subjected to SDS-PAGE. The Relia blot system (GE Healthcare, Buckinghamshire, UK) was used to detect the immune reactive bands by Kodak X-ray film (Kodak, Tokyo, Japan).

### RNA Extraction and RT-PCR

RNA extraction and RT-PCR analysis of mRNAs for ATGL and PPARg-2 and b-actin were performed using an RT-PCR system, as previously described [47], [48]. Twenty-five cycles of amplification were carried out for ATGL and PPARg-2 mRNAs, and 20 cycles were used for b-actin mRNA. The conditions of each cycle were denaturated at 94°C for 10 sec, annealed at 55°C for 10 sec, and extended at 72°C for 30 sec. The primers are described below:

ATGL: 5′- AGTTCAACCTTCGCAATCTC-3′(sense),

5′-GTCACCCAATTTCCTCTTGG -3′(antisense).

PPARg-2∶5′-ACTGCCTATGAGCACTTCAC-3′ (sense),

5′-GATGGCATTGTGAGACATCC-3′ (antisense).

b-actin: 5′-ACCTGACAGACTACCTCATG-3′ (sense).

5-ACTCATCGTACTCCTGCTTG-3′ (antisense).

### Measurement of Plasma Insulin Levels

Blood samples were collected directly from the abdominal aorta into 24-gauge needles treated with heparin sodium solution and attached to a syringe. Plasma samples were separated via centrifugation at 6,000 *g* and 4°C for 15 min, and then stored at −80°C until analysis. Plasma insulin was determined using an enzyme-linked immunosorbent assay kit, according to protocol (Shibayagi Co. Ltd., Gumma, Japan).

### Statistical Analysis

Values represent the means ± S.D. The significance of differences between means was assessed using the Scheffe’s test after the analysis of variance had been performed to establish that there were significant differences between the groups. P<0.05 was regarded as significant.
